# Blood substitution therapy rescues the brain of mice from ischemic damage

**DOI:** 10.1038/s41467-020-17930-x

**Published:** 2020-08-25

**Authors:** Xuefang Ren, Heng Hu, Imran Farooqi, James W. Simpkins

**Affiliations:** 1 Department of Neuroscience, West Virginia University, Morgantown, West Virginia 26506 USA; 2 Department of Microbiology, Immunology and Cell Biology, School of Medicine, West Virginia University, Morgantown, West Virginia 26506 USA; 3 Experimental Stroke Core, Center for Basic and Translational Stroke Research, Rockefeller Neuroscience Institute, West Virginia University, Morgantown, West Virginia 26506 USA; 4 Present Address: Erma Building Room 109, 64 Medical Center Drive, Morgantown, West Virginia 26506-9303 USA; 5 Present Address: 64 Medical Center Drive, Morgantown, West Virginia 26506-9303 USA; 6 Present Address: EWMS Physical Medicine and Rehabilitation, 721 Fairfax Avenue, 3rd Floor, Norfolk, Virginia 23507-2007 USA; 7 Present Address: Erma Building Room 105, 64 Medical Center Drive, Morgantown, West Virginia 26506-9303 USA

**Keywords:** Neuroimmunology, Stroke

## Abstract

Acute stroke causes complex, pathological, and systemic responses that have not been treatable by any single medication. In this study, using a murine transient middle cerebral artery occlusion stroke model, a novel therapeutic strategy is proposed, where blood replacement (BR) robustly reduces infarctions and improves neurological deficits in mice. Our analyses of immune cell subsets suggest that BR therapy substantially decreases neutrophils in blood following a stroke. Electrochemiluminescence detection demonstrates that BR therapy reduces cytokine storm in plasma and ELISA demonstrates reduced levels of matrix metalloproteinase-9 (MMP-9) in the plasma and brains at different time points post-stroke. Further, we have demonstrated that the addition of MMP-9 to the blood diminishes the protective effect of the BR therapy. Our study is the first to show that BR therapy leads to profoundly improved stroke outcomes in mice and that the improved outcomes are mediated via MMP-9. These results offer new insights into the mechanisms of stroke damage.

## Abstract (web summary)

Acute stroke causes complex, pathological, and systemic responses which remain challenging to treat. Here, the authors show that substituting the blood of stroke model mice with whole-blood from naive healthy donor mice reduces infarct volume and improves neurological deficits.

## Introduction

A stroke remains a major cause of morbidity and mortality globally^1^. Current treatments for acute stroke include thrombolytic therapy through the administration of tissue plasminogen activator and the surgical removal of the clot. However, these methods have limited time windows. In the clinical field of stroke, the mantra is “time is brain,” because infarct evolves every minute following a stroke.

A stroke is more than just a disruption of blood flow to the brain; stroke pathophysiology is a progressive and systemic response to brain injury^2^. Dynamic breaches of the blood–brain barrier (BBB) has been observed in experimental stroke animal models^3,4^ and stroke patients^5^. Following BBB disruption after ischemic stroke, a pathological and systemic reaction may be initiated. A hyperinflammatory condition, including an increase in inflammatory cells, cytokines, and chemokines in circulating blood has been documented in stroke^6^. Activated neutrophils secret proteinases such as matrix metalloproteinase-9 (MMP-9), which may cause BBB leakage, extracellular matrix degradation, and evolution of cerebral ischemia^7^. MMP-9 also interacts with chemokines^8^ and cytokines^9–11^, causing a further cascade of post-ischemic cerebral inflammation that leads to degeneration in brain tissue and exacerbates stroke outcomes.

In this study, using a murine transient middle cerebral artery occlusion (tMCAO) stroke model, we present a therapeutic strategy for stroke—a blood replacement (BR) that substitutes stroke mouse blood with whole blood obtained from naive, healthy donor mice. We have demonstrated that the BR performed at 6.5~7 h following a stroke robustly reduces infarct volume and improves neurological deficits. Following the treatment, we demonstrate that the BR therapy significantly reduces the cytokine storm in the plasma. In addition, flow cytometry and enzyme-linked immunosorbent assay (ELISA) have demonstrated substantially reduced neutrophils and decreased levels of MMP-9 in the blood and brains of stroke mice, which received whole blood obtained from naive donor mice. Further, addition of MMP-9 in blood diminishes therapeutic effects of the BR therapy. These results reveal a possible therapy of using blood for brain protection from a stroke.

## Results

### The BR therapy improves stroke outcomes in post-stroke mice

Using an experimental stroke animal model of tMCAO, we demonstrated that infarction evolves following a 90 min tMCAO and reperfusion (Supplementary Fig. 1). We determined larger infarction from stroke mice at 23 h compared to 6 h, indicating that the injury evolves over time after removals of filaments in MCA-occluded stroke mice. We have also demonstrated that stroke alters cellular profiles in blood following a 90 min tMCAO and ischemic reperfusion in mice (Supplementary Fig. 2). We found that absolute cell numbers of neutrophils (Gr1^+^), monocytes, CD4^+^ cells, CD8^+^ cells, and NK1.1^+^ cells were significantly increased but CD19^+^ B cells were decreased at 6 h post stroke compared to controls. The data suggest that blood might play a critical role in the evolution of a stroke and the injured brain may be rescued if a blood-based strategy is used during a critical time window.

**Fig. 1:**
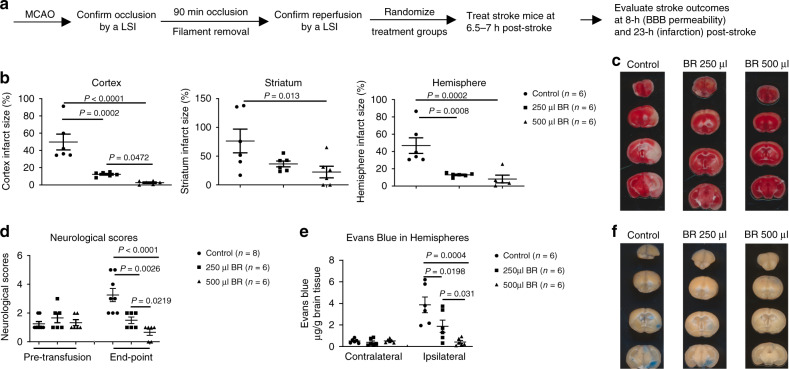
Blood replacement improves stroke outcomes. **a** Experimental design. Mice (8~12 months old males) underwent tMCAO for 90 min. The occlusion and reperfusion were confirmed by a laser speckle imager (LSI). Mice were randomized into three groups: sham control, 250 µl, or 500 µl of blood from healthy donors (3~6 months old). Blood was transfused into stroke mice at 6.5~7 h post stroke. The same volume of blood was withdrawn from the recipient stroke mice during blood transfusion. Brain infarct volumes were measured at 23 h after ischemia induction. BBB permeability was evaluated at 1 h post blood replacement (BR). **b** BR reduced infarct volume in the cortex, striatum, and total hemisphere dose dependently. Sham control (circles, *n* = 6), 250 µl group (squares, *n* = 6), or 500 µl group (triangles, *n* = 6) of blood from healthy donors. **c** Representative triphenyltetrazolium chloride (TTC)-stained coronal brain sections used for infarction analyses. **d** Neurological deficits were significantly improved in the BR group. Sham control (circles, *n* = 8), 250 µl group (squares, *n* = 6), or 500 µl group (triangles, *n* = 6) of blood from healthy donors. **e** BR reduced Evans blue extravasation in the brain dose dependently. Sham control (circles, *n* = 6), 250 µl group (squares, *n* = 6), or 500 µl group (triangles, *n* = 6) of blood from healthy donors. **f** Representative coronal brain sections showing Evans blue extravasation. Data were presented as means ± SEM. One-way ANOVA followed by post hoc Fisher’s unprotected least-significant difference for multiple comparison tests. Source data are provided as a Source Data file.

To determine the role of blood in stroke, we conducted a dose–response experiment of BR on stroke outcomes using a murine tMCAO model (Fig. 1a). Before the BR, we recorded global cerebral blood flow (CBF) changes at four time points as follows: prior to tMCAO, at 1 min post tMCAO, at 5 min post reperfusion, and prior to the BR in mice using a Laser Speckle Imager. We confirmed that the MCAO reduced 70~80% relative CBF (ratio of CBF in the right MCA territory : CBF in the left MCA territory) at the post-tMCAO time point and reperfusion fully recovered the relative CBF at 5 min post-reperfusion measurements and prior to the BR in all stroke mice; however, no significant differences were detected among randomized groups of mice (Supplementary Fig. 3). Then, we replaced stroke mice blood with different doses of whole blood obtained from naive donor mice at 6.5~7 h after ischemia. Although the data demonstrated that 250 and 500 µl of the blood robustly reduced stroke infarction in the cortex, striatum, and hemisphere compared to sham control group, 500 µl of the blood had a greater protection in brain damage following a stroke (Fig. 1b, c). Interestingly, neurological deficits were significantly improved in the BR groups compared to the control group (Fig. 1d). Furthermore, we performed an Evans blue extravasation assay and demonstrated that 250 and 500 µl of blood protected the BBB, as evidenced by less Evans blue extravasation in ischemic hemispheres (Fig. 1e, f). However, the BR therapy did not change physiology parameters in the blood of stroke mice (Supplementary Table 1).

**Fig. 2:**
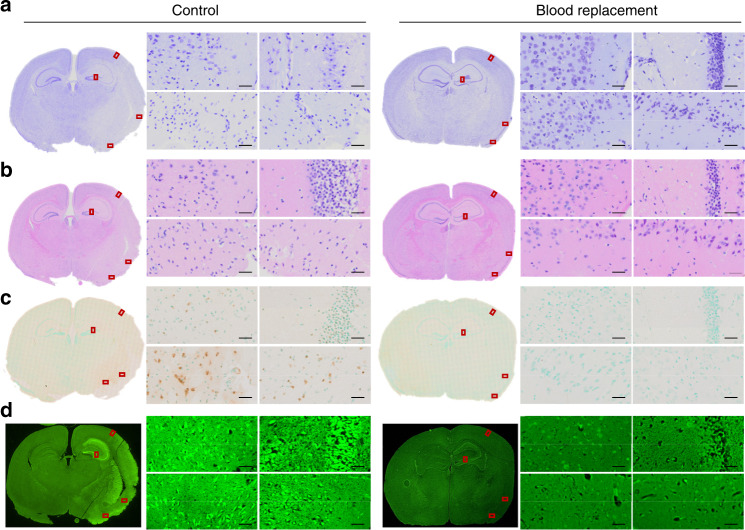
Blood replacement protects the brain from cell death in stroke mice. Microscopic images of brain tissue taken from stroke mice underwent tMCAO for 90 min followed by 22.5 h reperfusion. The blood replacement (BR) group received 500 µl of blood from healthy donors (3~6 months old) at 6.5~7 h post stroke. **a** The Cresyl violet staining, **b** H&E staining, **c** TUNEL staining counterstained with Methyl Green, and **d** Fluoro-Jade C staining were performed on paraffin embedded brain slices. Red rectangles indicate the magnified areas evaluated under ×40 magnification. Both the cortex and striatum were evaluated. Experiments were repeated five times with similar results and images were presentative of one mouse from five mice per group. Scale bars = 20 µm.

To further confirm the pathological changes in stroke brains, we conducted cresyl violet staining (Fig. 2a) and hematoxylin and eosin (H&E) staining (Fig. 2b). At high magnification, we observed typical pyknotic neurons or dark neurons in selected cortex and striatum areas in control mice following 90 min tMCAO at 23 h post stroke. Interestingly, morphologically intact neurons were broadly seen in similar brain areas of mice with the BR therapy. The degenerating neurons were broadly detectable by terminal deoxynucleotidyl transferase dUTP nick end labeling (TUNEL) staining (Fig. 2d, positive staining in brown) and Fluora-Jade C (Fig. 2d, positive staining in fluorescent green) staining in control mice but only a few in BR-treated mice. The data clearly demonstrated that the BR therapy prevent brain cells from ischemic damage.

**Fig. 3:**
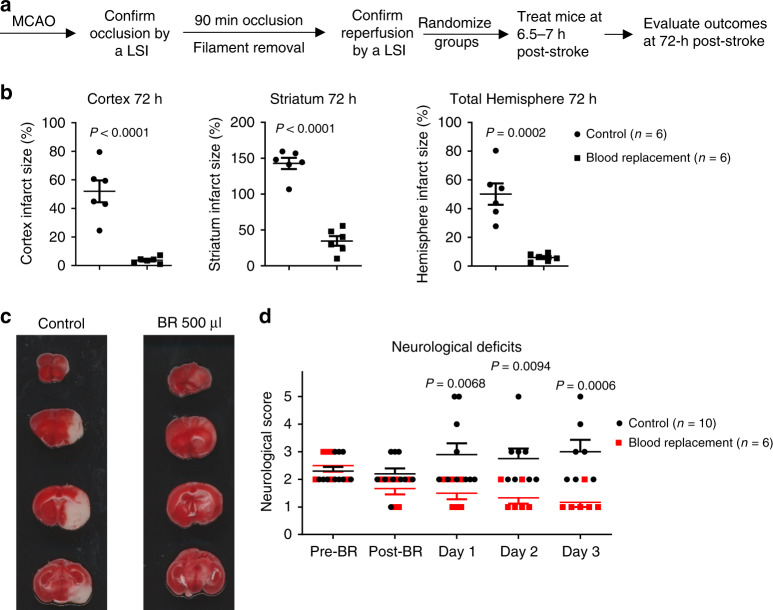
Blood replacement therapy protects infarct evolution. **a** Experimental design. Mice underwent tMCAO for 90 min. The occlusion and reperfusion were confirmed by laser speckle imager (LSI). Mice were randomized into two groups: sham controls and the blood replacement (BR) group receiving 500 µl of blood from healthy donors (3~6 months old). Blood was transfused into stroke mice at 6.5~7 h post stroke. The same volume of blood was withdrawn from the recipient stroke mice during blood transfusion. Brain infarct volumes were measured at 72 h after ischemia induction. **b** BR therapy reduced infarct volume in the cortex, striatum, and total hemisphere. Sham controls (circles, *n* = 6) and the BR group (squares, *n* = 6). **c** Representative TTC-stained coronal brain sections used for infarction analyses. **d** Neurological deficits were significantly improved in the BR group from day 1 to day 3. Sham controls (circles, *n* = 10) and the BR group (squares, *n* = 6). Data were presented as means ± SEM; *P*-values were calculated using two-tailed grouped analyses by Student’s *t*-test. Source data are provided as a Source Data file.

Then we ask whether the BR is neuroprotective or just delays the eventual infarct evolution to its final state. We treated stroke mice with the BR therapy and then evaluated outcomes at 72 h post stroke (Fig. 3). Infarction did not further evolve in BR therapy-treated stroke mice, while matured infarction was observed in control mice by TTC staining (Fig. 3b, c). The BR therapy-treated mice had significantly better functional outcomes than control mice from post-stroke day 1 through day 3 (Fig. 3d). Taken together, these data suggest that BR therapy results in a profound protection in ischemic brains and offers a therapeutic effect for stroke.

We next sought to understand whether blood transfusion alone or blood withdrawal alone protects stroke outcomes. The mice in both blood transfusion alone and blood withdrawal alone groups had large infarction and high mortalities (six of nine mice died in each group within 23 h of stroke) (Supplementary Fig. 4), suggesting that transfusion of blood and withdrawal of blood are both required for BR therapy for stroke treatment.

To further assess the effects of blood on outcomes in stroke, we transfused 500 µl of whole blood obtained from stroke mice, but this cohort of mice had larger infarct volumes and a higher mortality (six of nine recipient stroke mice died within 23 h of stroke, mortality is 66.7%) than the cohort of mice replaced with blood obtained from naive donor mice (0 of six mice died) (Supplementary Fig. 5). Fisher’s exact test determined 90% power of the mortality differences between whole blood obtained from stroke mice and naive donor mice. The data suggest that blood from healthy donors is important when applying BR therapy in stroke.

### The BR therapy alters inflammatory responses in the periphery of stroke mice

**Fig. 4:**
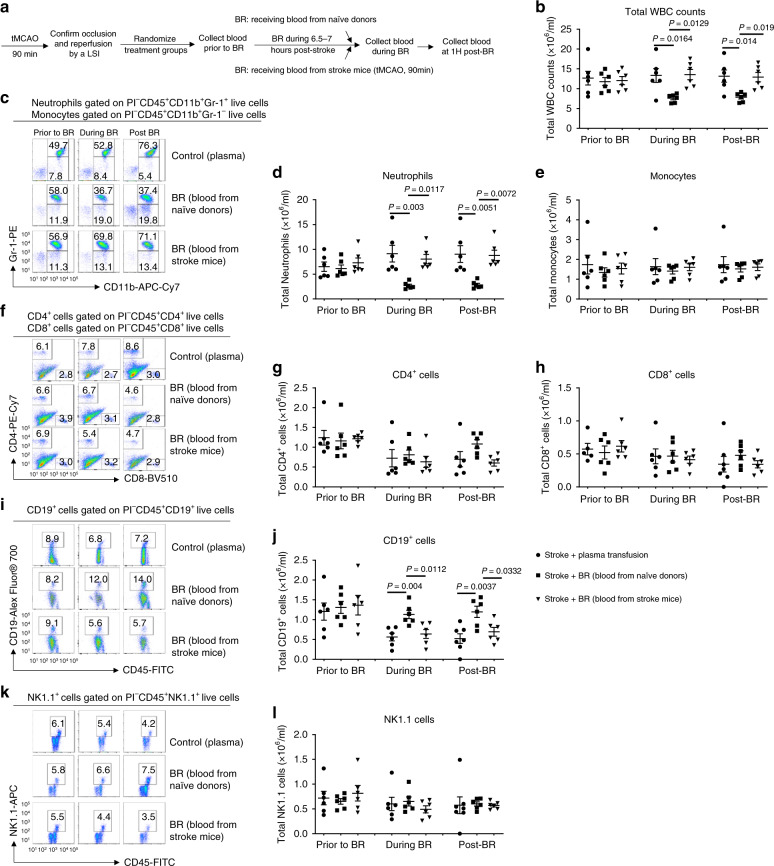
Blood replacement therapy alters cellular profiles in blood of stroke mice. **a** Experimental design. Mice (8 months old males) underwent tMCAO for 90 min. The occlusion and reperfusion were confirmed by laser speckle imager (LSI). Mice were randomized into three groups: receiving 500 µl of plasma (circles, *n* = 6) from healthy donors, 500 µl of blood from healthy donors (squares, *n* = 6), and 500 µl of blood from stroke mice (90 min tMCAO followed by 4.5 h reperfusion) (triangles, *n* = 6). Blood was transfused into stroke mice at 6~8 h post stroke. The same volume of blood was withdrawn from the recipient stroke mice during blood transfusion. Blood obtained from recipient stroke mice were analyzed at three time points: prior to blood replacement (BR), during BR, and at 1 h post BR. **b** Total white blood cells were counted. **c** Representative flow cytometry images for neutrophils gated on propidium iodide (PI)^−^CD45^+^CD11b^+^Gr-1^+^ populations and monocytes gated on PI^-^CD45^+^CD11b^+^Gr-1^−^ populations. **d** Total neutrophil counts. **e** Total monocyte counts. **f** Representative flow cytometry images for CD4^+^ cells gated on PI^−^CD45^+^CD4^+^ populations and CD8^+^ cells gated on PI^−^CD45^+^CD8^+^ populations. **g** Total CD4^+^ cell counts. **h** Total CD8^+^ cell counts. **i** Representative flow cytometry images for CD19^+^ B-cells gated on PI^−^CD45^+^CD19^+^ populations. **j** Total CD19^+^ B-cell counts. **k** Representative flow cytometry images for NK1.1^+^ cells gated on PI^−^CD45^+^NK1.1^+^ populations. **l** Total NK1.1^+^ cell counts. Data were presented as means ± SEM. One-way ANOVA followed by post hoc Tukey’s test was used to determine the significance. Source data are provided as a Source Data file.

It is now accepted that inflammation and the immune system are critical in acute stroke damage^12,13^. As shown in Supplementary Fig. 2, stroke significantly changed cellular profiles in the blood of mice following tMCAO. To further investigate how BR therapy affects the cellular populations in the blood of stroke mice, we assessed cellular subsets in the blood of recipient stroke mice at three time points—prior to BR, during BR, and at 1 h post BR. The recipient stroke mice received 500 µl of whole blood obtained from either naive donors or stroke mice and additional control group received 500 µl of plasma obtained from naive donors (Fig. 4a). In randomized groups, total white blood cell counts were not significantly different prior to the BR (Fig. 4b). Interestingly, whole blood obtained from naive donors resulted in significantly lower total leukocyte numbers and neutrophils during BR and at 1 h post BR in the blood of recipient stroke mice compared to whole blood obtained from stroke mice or plasma obtained from naive donors. There were substantially more total leukocytes and neutrophils but less CD19^+^ B cells in the blood of recipient stroke mice that received whole blood from the stroke mice group and plasma from the naive donor group than the mice that received whole blood from naive donor group. There were no significant changes in monocytes CD4^+^ cells, CD8^+^ cells, and NK1.1^+^ cells in blood of recipient stroke mice among plasma from naive donor group, whole blood from naive donor group, and whole blood from the stroke mice group (Fig. 4c–l).

**Fig. 5:**
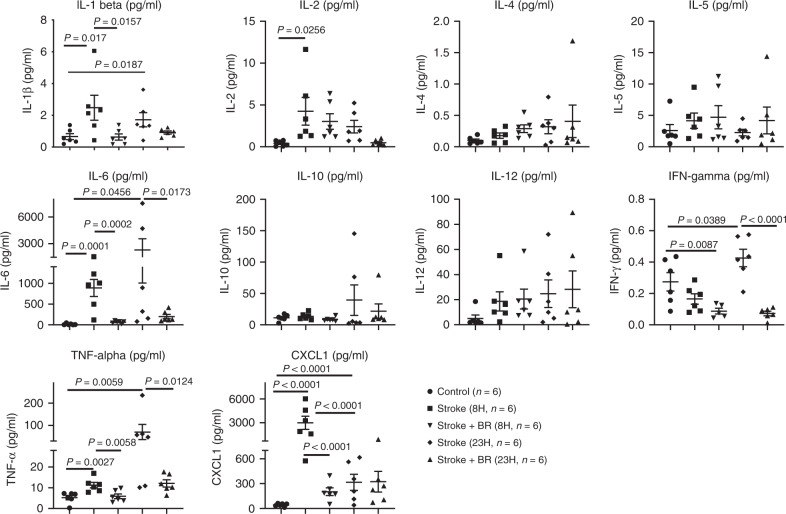
Blood replacement therapy alters cytokine panel in the plasma of stroke mice. Naive mice (8~12 months old males) serve as a control group (circles, *n* = 6). Stroke mice (8~12 months old males) underwent tMCAO for 90 min. The occlusion and reperfusion were confirmed by a laser speckle imager. Stroke mice were then randomized into four groups: two groups being killed at 8 (squares, *n* = 6) and 23 h (diamonds, *n* = 6) post stroke; two groups receiving blood replacement (BR) therapy and being killed at 8 (down-pointing triangles, *n* = 6) and 23 h (up-pointing triangles, *n* = 6) post stroke. Then plasma was collected for the analyses of proinflammatory cytokine panel including IL-1β, IL-2, IL-4, IL-5, IL-6, IL-10, IL-12, IFN-γ, TNF-α, and the chemokine, CXCL1. Data were presented as means ± SEM. One-way ANOVA followed by post hoc Fisher’s unprotected least-significant difference for multiple comparison tests. Source data are provided as a Source Data file.

In line with the changes of cell numbers in blood of stroke mice, the levels of proinflammatory cytokines and the chemokine, CXCL1, in plasma were also altered (Fig. 5). The data demonstrated that BR therapy significantly reduced the levels of proinflammatory cytokines interleukin (IL)-1β, IL-6, TNF-α, and the chemokine, CXCL1, at 8 h post stroke and further reduced IL-6, IFN-γ, and TNF-α at 23 h post stroke. CXCL1 known as a neutrophil-activating protein is responsible for neutrophil chemotaxis^14^. The significant change of CXCL1 at early stroke suggests that neutrophils might have played an important role in stroke and involved in BR therapy.

### The BR therapy alters cerebral leukocyte invasion in stroke mice

**Fig. 6:**
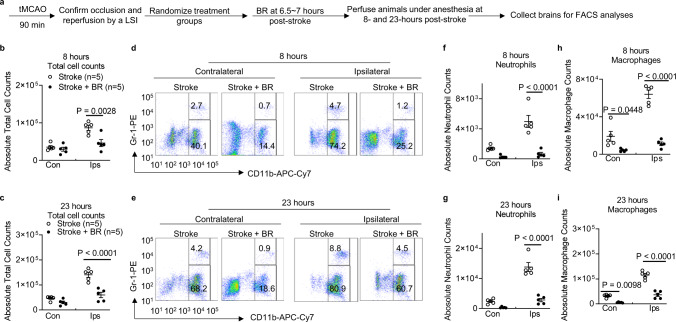
Blood replacement therapy reduces cell infiltration into brains of stroke mice. **a** Experimental design. Mice (8~12 months old males) underwent tMCAO for 90 min. The occlusion and reperfusion were confirmed by a laser speckle imager (LSI). Recipient mice were then randomized into two groups: a control group receiving sham surgery (white circles, *n* = 5) and a blood replacement (BR) group receiving 500 µl of blood from healthy donors (black circles, *n* = 5). Blood was replaced into stroke mice at 6.5~7 h post stroke. The same volume of blood was withdrawn from the recipient stroke mice during blood transfusion. Brains were perfused and analyzed at 8 and 23 h after ischemia induction. Blood from naive donors reduced numbers of total inflammatory cells in ischemic hemispheres at **b** 8 and **c** 23 h post stroke. Representative flow cytometry images for neutrophils gated on propidium iodide (PI)^−^CD45^+^CD11b^+^Gr-1^+^ populations and macrophages gated on PI^−^CD45^+^CD11b^+^Gr-1^−^ populations in hemispheres at **d** 8 and **e** 23 h post stroke. Blood from naive donors reduced numbers of absolute neutrophil counts in ischemic hemispheres at **f** 8 and **g** 23 h post stroke. Blood from naive donors reduced numbers of absolute macrophage counts in ischemic hemispheres at **h** 8 and **i** 23 h post stroke. Data were presented as means ± SEM. One-way ANOVA followed by post hoc Tukey’s tests. Source data are provided as a Source Data file.

Leukocytes are the major effectors of inflammatory damage in stroke brains. Among them, neutrophils and macrophages are early responders to a stroke. To investigate whether BR therapy affects central nervous system (CNS) inflammatory infiltration, we designed a study to analyze leukocyte invasion in the hemispheres of stroke mice (Fig. 6a). The BR therapy significantly reduced total absolute cell numbers in ischemic hemispheres at 8 and 23 h post stroke (Fig. 6b, c). To further investigate how BR therapy affects leukocyte composition in the brain after stroke, numbers of infiltrating Gr1^+^ neutrophils, and CD11b^+^CD45^high^ macrophages, CD3^+^ T cells and CD19^+^ B cells were assessed by flow cytometry. At both time points, the accumulation of neutrophils and macrophages was significantly less in the affected hemispheres of BR-treated stroke mice compared with control stroke mice (Fig. 6d–i). These data suggest that BR therapy can reduce CNS inflammatory responses to a stroke.

### MMP-9 is involved in the BR therapeutic in stroke

**Fig. 7:**
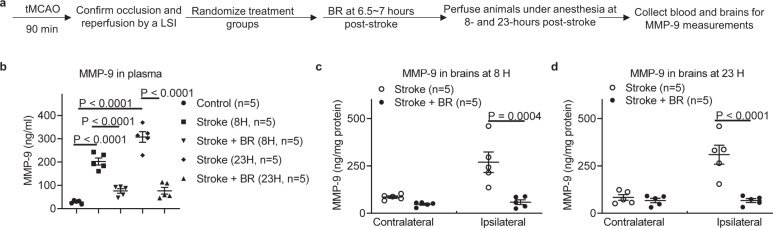
Blood replacement therapy reduces levels of MMP-9 in the plasma and brains in stroke mice. **a** Experimental design. Mice (8~12-month-old males) underwent tMCAO for 90 min. The occlusion and reperfusion were confirmed by a laser speckle imager (LSI). Stroke mice were then randomized into two groups: a stroke only group and a blood replacement (BR) therapy group receiving 500 µl of blood from healthy donors. Blood was replaced into stroke mice at 6.5~7 h post stroke. Brains were perfused and analyzed at 8 and 23 h after ischemia induction. **b** BR therapy reduced levels of MMP-9 in the plasma at 8 and 23 h post stroke. Control (circles, *n* = 5), stroke mice analyzed at 8 h post stroke (squares, *n* = 5), stroke mice with BR therapy analyzed at 8 h post stroke (down-pointing triangles, *n* = 5), stroke mice analyzed at 23 h post stroke (diamonds, *n* = 5), stroke mice with BR therapy analyzed at 23 h post stroke (up-pointing triangles, *n* = 5). The BR therapy reduced the levels of MMP-9 in ischemic hemispheres at **c** 8 and **d** 23 h post stroke. Data were presented as means ± SEM. One-way ANOVA followed by post hoc Tukey’s tests. Stroke mice group (white circles, *n* = 5); stroke mice with BR therapy group (black circles, *n* = 5). Source data are provided as a Source Data file.

MMP-9 is one of proteinases that is expressed by neutrophils and macrophages/monocytes^15^. MMP-9 is increased in the blood within the first 2–6 h of stroke in patients, primates, and rodents^16–19^. Given that BR therapy robustly reduced neutrophil counts in the peripheral blood and significantly decreased the infiltration of neutrophils and macrophages/monocytes in ischemic hemispheres, it is important to further investigate how MMP-9 is involved in BR-treated stroke. We designed a study to evaluate the levels of MMP-9 in the plasma and brains following tMCAO with the BR therapy compared to controls (Fig. 7a). We demonstrated increased levels of MMP-9 in the plasma obtained from tMCAO mice compared to controls at both 8 and 23 h post stroke (Fig. 7b). Interestingly, BR therapy remarkably reduced the levels of MMP-9 in the plasma of recipient stroke mice at both early and late time points (Fig. 7b). However, MMP-2 was not significantly changed between two groups at either time points (Supplementary Fig. 6). We further evaluated the levels of MMP-9 in the brains of these mice and demonstrated that BR therapy significantly reduced the levels of MMP-9 in ischemic hemispheres as well (Fig. 7c, d). These data are consistent with neutrophil changes in the periphery and CNS (Figs. 4 and 6), and suggest that the decreased level of MMP-9 could be one of the mechanisms by which the BR therapy improves stroke outcomes.

**Fig. 8:**
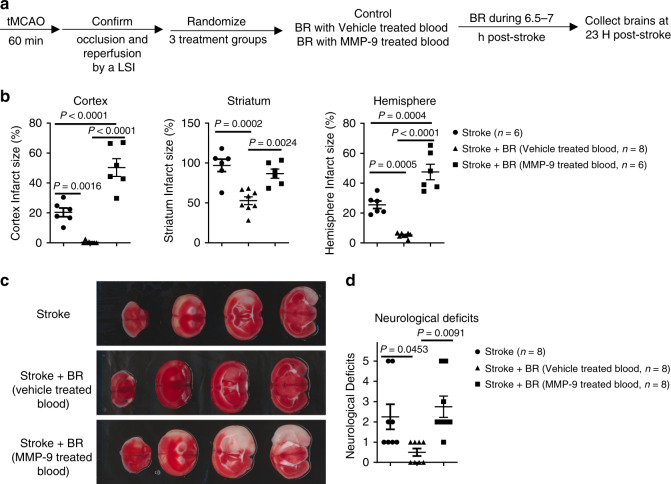
MMP-9 diminished the therapeutic effects of the blood replacement in stroke mice. **a** Experimental design. Mice (8-month-old males) underwent tMCAO for 60 min. The occlusion and reperfusion were confirmed by a laser speckle imager (LSI). Mice were randomized into three groups: the first group is the stroke group received tMCAO, the second group is the blood replacement (BR) group receiving 250 µl of blood with vehicle and blood withdrawal, and the third group is the BR group receiving 250 µl of blood treated with activated MMP-9 (5 µg ml^−1^) and blood withdrawal. **b** Quantification of infarct volumes in the cortex, striatum, and total hemisphere. MMP-9-treated blood did not significantly reduce the infarct volume. Stroke group (circles, *n* = 6), stroke with vehicle-treated BR group (triangles, *n* = 8), and stroke with MMP-9-treated BR group (squares, *n* = 6). **c** Representative TTC-stained coronal brain sections used for infarction analyses. **d** Neurological deficits were not significantly improved in the BR group with MMP-9-treated blood. Stroke group (circles, *n* = 8), stroke with vehicle-treated BR group (triangles, *n* = 8), and stroke with MMP-9-treated BR group (squares, *n* = 8). Data were presented as means ± SEM. One-way ANOVA followed by post hoc Tukey’s multiple comparison tests. Source data are provided as a Source Data file.

To further investigate the role of MMP-9 in the BR therapy in stroke, we designed a study of using MMP-9-treated blood compared to vehicle-treated blood for transfusion (Fig. 8a). To compromise the high mortality from 90 tMCAO mice, a 60 min tMCAO protocol is applied to the study. Interestingly, MMP-9-treated blood significantly exacerbated stroke infarction and worsened neurological deficits, whereas vehicle-treated blood profoundly protected stroke outcomes following 60 min tMCAO and 22 h reperfusion (Fig. 8c, d). The data clearly demonstrated that MMP-9 diminished the therapeutic effects of BR in stroke and suggested that a reduced level of MMP-9 could be one of the important mechanisms used by the BR therapy in stroke.

## Discussion

There may be multiple mechanisms occurring in BR. First, brain antigens are released through the BBB openings and may activate the immune system after stroke. Thus, the removal of blood from stroke mice may reduce the levels of brain antigens in circulating blood and alleviate the activation of immune system by brain antigens. Second, the removal of blood from stroke mice may diminish activated white blood cells and decrease many deleterious signals, such as cytokines, chemokines, proteases, and proteinases in circulating blood after stroke. Third, as observed in patients with thrombotic thrombocytopenic purpura (TTP) disease, where the process of a blood transfusion may supply nutrition factors for the TTP patients^20^, blood may also provide oxygen and many other neuroprotective factors for stroke brains. The mechanisms in the BR therapy for stroke may be more complicated than one factor and it could be a challenge to address all mechanisms here. We here demonstrate the BR therapy as an effective therapeutic strategy for stroke treatment and elucidate MMP-9 involved mechanism in the therapy.

The average total blood volume of a mouse is about 77~80 ml kg^−1^ (0.077 ~ 0.080 ml gram^−1^)^21^. A mouse weighing 30 g would therefore have a total blood volume of ~(77~80) ml kg^−1^ × 0.03 kg = 2.31 ~ 2.4 ml. Transfusion of 500 µl of blood is in <20% total blood volume. This volume is translatable to human for blood transfusion.

We have investigated the role of separated blood components by the BR treatment in stroke. We conducted a study by testing the effects of transfusion up to 500 µl of plasma while withdrawing the same amount of blood from stroke mice. It has been demonstrated that plasma from young mice ameliorates pathology and cognition in a mouse model for Alzheimer’s disease^22^. However, the results demonstrated that plasma transfusion did not protect stroke outcomes as observed in whole-BR (Supplementary Fig. 7). Plasma transfusion did not change cellular components in blood as compared to whole blood obtained from stroke mice donors (Fig. 4). The data also suggest that the beneficial effects of the BR therapy is not simply due to a dilution of blood in recipients. Transfusion of 500 µl of whole blood or plasma is considered less than 20% of total blood volume into a mouse. However, reduced neutrophil numbers (Fig. 4b) and the levels of MMP-9 (Fig. 7b) by the BR therapy are far more than 20%. In addition, the data from proinflammatory cytokine and chemokine panel (Fig. 7) also suggest that BR therapy does not simply cause a dilution effect in the blood of stroke mice. Therefore, we believe that BR therapy might reduce a cascade response to a stroke that may be able to provide such a profound protection in stroke brains.

It is important to identify key factors responsible for the positive effects of a blood transfusion, because they may lead to the development of cellular and molecule interventions with a better efficacy. However, the role of specific cell types in producing ischemic brain damage remains a controversial topic and the mechanisms of how inflammatory cells and their mediators are involved in stroke-induced neuro-inflammation are still not fully understood. In patients with ischemic stroke, the number of circulating neutrophils rise within the first few hours of stroke onset^23^. This increase is associated with stroke severity^24^, infarct volume^25^, and worse functional outcomes^26,27^. Kleinschnitz et al.^28^ has demonstrated that transfer of CD3^+^ T cells is detrimental for cerebral ischemia and T cells critically contribute to cerebral ischemia; however, the role of regulatory T cells has been a controversial concept in the stroke field for a decade^29^. B cells are documented protective in acute stroke^30^. Although we determined that the BR therapy substantially decreased neutrophils in blood (Fig. 4c, d) and brains (Fig. 6) of stroke mice, we demonstrated that the removal of blood from recipient stroke mice is essential to develop this therapeutic strategy (Supplementary Fig. 4), which may help release of inflammatory cells from blood. We argue that the BR therapy could change leukocyte trafficking and migration into brains when the BBB is closed. For example, macrophages/monocytes were not significantly changed in the peripheral blood (Fig. 4c, e) but BR therapy substantially decreased macrophages/monocytes in stroke brains; B cells were significantly increased in the peripheral blood by the BR therapy (Fig. 4i, j) but very few B cells were detected in ischemic brains. It is important to determine specific roles of white blood cell subsets by BR therapy administration following a stroke to optimize therapeutic strategies in the future.

It has been confirmed that MMP-9 is a key proteinase associated with BBB leakage, extracellular matrix degradation, and evolution of cerebral ischemia^7^. Opening of the BBB is transient following a stroke^3^. Compromised BBB integrity allows blood solutes and inflammatory cells to flow into the brain causing adverse stroke outcomes such as brain edema and neuronal death. The shorter time the BBB remains open, the less influx of deleterious materials enters into the brain. We did observe that the BBB is protected following the BR therapy in stroke mice (Fig. 1e, f). Decreased level of MMP-9 in the periphery and CNS (Fig. 7) could be one important mechanism in closing the BBB by BR therapy for stroke. We have recently demonstrated a mitochondrial mechanism in BBB opening following a stroke^31–33^. Due to the analyses of physiological parameters demonstrated that BR therapy did not change PO2, PCO2, and glucose level in recipient stroke mice (Supplementary Table 1), our data do not support the possibility that BR therapy rescues ischemic brains by changing the physiological parameters. However, it is possible that blood provides nutrition and protective factors that can help cerebrovascular endothelial cells restore mitochondrial function and seal BBB tight junctions following a stroke.

It is known that inflammatory cytokines and chemokines play a harmful role in acute stroke^34^. We observed significantly higher levels of proinflammatory cytokines IL-1β, IL-6, and TNF-α in plasma of stroke mice and the BR therapy decreased the levels of these cytokines in plasma (Fig. 5). As active MMP-9 cleaves a variety of chemokines^8^ and cytokines^9–11^, and triggers signaling through various transmembrane proteins^35^, the BR therapy might reduce the cascade inflammatory responses in stroke through reduced levels of MMP-9 in blood (Fig. 7). It is noted that chemokine CXCL1, a neutrophil-activating protein, is substantially increased in stroke and significantly reduced by the BR therapy at an early time point post stroke. The cellular source of CXCL1 is not clear; however, the elevated CXCL1 may explain that neutrophils are the first responders that react to acute ischemia^36^ and CXCL1 may account for the rise of neutrophils in the blood and brains following a stroke.

Others have demonstrated that knockout of MMP-9 or inhibition of MMP-9 can be of therapeutic importance in ischemic stroke^37^. It has been shown that MMP-9 inhibitors DP-b99^38^, KB-R7785^39^, SB-3CT^40^, and BB-94^37^ protect stroke outcomes. Even though BR therapy profoundly protects stroke outcomes, addition of MMP-9 to the blood diminishes the protective effect of the BR therapy and substantially worsens stroke outcomes (Fig. 8). These data elucidate an MMP-9-involved mechanism in the BR therapy in stroke and also suggest the importance of measuring the level of MMP-9 in donor blood for transfusion. The dose of MMP-9 administrated is 1.25 µg 30 g^−1^ mouse, relative to 500 ng ml^−1^ MMP-9 in blood of recipient mice. This level corresponds to doubled levels of MMP-9 in blood following a stroke (Fig. 7a). It is reported that higher plasma levels of pro-MMP-9 early in stroke are correlated with larger infarct volumes^41^. We did observe that longer occlusion causes worse stroke outcomes and more proinflammatory responses in early stroke. Permanent stroke causes more profound inflammatory responses and higher levels of MMP-9 in blood. Therefore, blood obtained from permanent stroke mice donors worsened stroke outcomes and caused a high mortality in stroke mice (Supplementary Fig. 5).

Sex differences exist in response to stroke^42–45^ and age is another factor that affects stroke outcomes^46^. It is not clear whether blood obtained from different sexes and ages contributes to different outcomes in stroke. We used blood obtained from relatively young mice (3~6 months old) and transfused the whole blood into older stroke mice (8~12 months old) in the study. Our ongoing study will further address the role of blood obtained from a wide range of ages of both males and females.

Currently, blood-based therapies are emerging as treatments to combat aging and fight neurodegenerative diseases^47^. It is not likely that the complex pathological changes following a stroke can be treated by any known, single medication. The proposed BR therapy offers a strategy that targets the pathological systemic responses to stroke. The results of this study could lead to a breakthrough in stroke therapy, because this innovative therapeutic strategy may reduce the mortality of stroke patients and improve stroke outcomes. These results can provide a foundation for the future use of BR therapy in clinical trials for improved treatment of strokes.

## Methods

### Study approval

The ARRIVE guidelines were followed and Institutional Animal Care and Use Committee at West Virginia University (WVU) approved criteria for procedures prior to experimentation.

*Animals*: We used male 8~12 months old C57/BL6J mice (above 30 g; Jackson’s Laboratory, ME) for recipients and male 3~6 months old C57/BL6J mice for blood donors. We used 333 mice for the study. We numbered the animals and allocated them into groups using a simple randomization of excel-generated random numbers. To avoid biases, we also assured that different treatments were performed on the same day.

### Murine focal cerebral ischemia model

We anesthetized mice with 4–5% isoflurane and maintained anesthesia such that animals did not respond to toe pinch with 1–2% isoflurane via face mask in oxygen-enriched air. We induced focal cerebral ischemia by occlusion of the right middle cerebral artery for 90 min with a 6.0 monofilament suture (Doccol, Sharon, MA) and maintained a rectal body temperature of 37 ± 0.5 °C throughout the surgery. We confirmed a successful occlusion by changes of regional cerebral blood flow using Laser Speckle Imager (Moor Instruments, UK) and applied a local analgesia (bupivacaine 1 mg kg^−1^, subcutaneously (s.c.)) after stroke surgeries.

### Preparation of blood for transfusion

We prepared fresh blood obtained from naive donors or stroke mice via cardiac puncture while animals were under deep anesthesia. We collected blood in a syringe containing citrate-phosphate-dextrose (CPD) buffer (150 µl ml^−1^ blood) and rapidly mixed with anti-coagulated CPD buffer for transfusion. Recombinant mouse MMP-9 protein (R&D, USA) was activated in buffer (50 mM Tris, 10 mM CaCl_2_, 150 mM NaCl, 0.05% Brij-35, pH 7.5) with 1 mM *p*-aminophenylmercuric acetate for 2 h then added in fresh prepared blood (5 µg ml^−1^) for transfusion.

### Blood replacement

We conducted all procedures under anesthesia (4–5% isoflurane induction and 1–2% isoflurane maintenance). We placed a catheter (pre-washed with CPD buffer and dried 26 G, Monoject^TM^, Covidien, Italy) into left femoral vein for blood transfusion and another catheter (pre-washed with heparin and dried) was placed into right femoral artery for the blood withdrawal. We connected a microinjection pump (Genie Touch^TM^, Lucca Technologies) with the catheter in the femoral vein with a speed of 20 µl min^−1^ for blood transfusion. To withdraw blood, we attached a syringe to the catheter in femoral artery. When the procedure was completed, we removed the catheters and blotted the incision with sterilized cotton swabs until cessation of bleeding. We then closed the incisions with sutures and applied a local analgesia (bupivacaine 1 mg kg^−1^, s.c.) following the BR surgeries.

### BBB permeability in vivo

We used Evan’s blue extravasation assay to evaluate BBB permeability in mice. We administered Evan’s blue intravenously through tail vein 30 minutes prior to euthanization. We transcardially perfused animals with saline and sectioned brains with a 2 mm coronal brain matrix. We weighed hemisphere samples and homogenized samples with 400 µL phosphate-buffered solution (PBS), and then precipitated with 50% trichloroacetic acid (Sigma, CA) overnight. We centrifuged all samples were 300 × *g* for 30 min to separate out the brain tissue in pellet prior to measuring. We quantified Evans blue in the brain by absorption at 610 nm with a plate reader (BioTek, Winooski, VT). Final results were quantified as Evans blue microgram/gram brain tissue.

### Exclusion criteria

We excluded animals according to the criteria below: (1) regional cerebral blood flow decreases <70% during occlusion as detected by Laser Speckle Imager; (2) surgical procedures last more than 30 min; (3) no neurological deficits at 3 h post-tMCAO; (4) invisible infarction in the MCA territory by TTC staining; (5) postmortem examination shows subarachnoid hemorrhage; and (6) substantial ambient temperature changes in the facility. Animals that died prior to the planned time of assessments were postmortem examined for subarachnoid hemorrhage and the mortality was recorded. In this study, five mice were excluded: three mice (one mouse in sham BR control group and two mice in BR group of blood obtained from stroke mice) because of subarachnoid hemorrhage, two mice (prior to group randomization), because the Laser Speckle Imager did not detect 70% reduction of CBF after MCAO. The experimenters were blinded to the treatments for data collection and analysis.

### Brain histology

We cut brains in 2 mm brain coronal matrix and stained resulting sections with 2% TTC (Sigma, Saint Louis, MO) in PBS at 37 °C for 30 min. We photographed brains using a photo scanner and fixed the tissues in 10% formalin for 1 week. We used four sections of seen images for data qualification. We analyzed infarct volumes within the cortex, striatum, and total hemisphere using NIH Image J software.

We embedded fixed brain sections in paraffin and cut coronal slices (10 µm) through the brain hippocampus area. We performed Cresyl Violet (Sigma, Saint Louis, MO), H&E (Sigma, Saint Louis, MO), TUNEL staining (TUNEL Assay Kit - HRP-DAB, Abcam, Cambridge, MA), and Fluoro-Jade C staining (Sigma, Saint Louis, MO) on slides following the manufacturers’ instructions. We took bright field images for Cresyl Violet, H&E, TUNEL staining, and fluorescent field images for Fuoro Jade C staining through a slide scanner (Olympus VS120, Japan) at the Microscope Imaging Facility at WVU.

### Neurological deficits

We measured neurological functioning on a 0–5 scale, where 0 = no neurological dysfunction; 1 = failure to extend contralateral forelimb when lifted by tail; 2 = circling to the contralateral side; 3 = falling to the contralateral side; 4 = nonspontaneous walk or in a comatose state; 5 = death.

### Flow cytometry

We lysed red blood cells in blood by red blood cell lysis buffer (eBioscience) and isolated leukocytes in brains by 37–70% Percoll (GE Healthcare) density gradient centrifugation. We counted cell numbers by a Coulter counter (Thermo Fisher). Cells were washed with buffer (PBS with 0.5% bovine serum abumin and 0.02% sodium azide) for three times and stained with antibodies listed in Supplementary Table 2. We used BD FACS LSRFortesa (12 fluorochromes, three-laser system, BD Biosciences) with FACS Diva version 8.0 software (BD Biosciences) to determine the phenotypes of leukocytes. We excluded dead cells by propidium iodide (Sigma, 20 µg ml^−1^ in PBS) positive staining and analyzed the data using Flowjo version 10 software (TreeStar).

### Electrochemiluminescence assay for cytokines and chemokines

We used an electrochemiluminescence assay to detect a proinflammatory panel by multiplex kits obtained from Meso Scale Discovery (Rockville, MD). The kits included individual assay for quantification of IFN-γ, IL-1β, IL-2, IL-4, IL-5, IL-6, IL-10, IL-12, and TNF-α, and the chemokine CXCL1. We followed the manufacturer’s protocol for evaluation of plasma samples. We read the results on MSD multi-array imaging platform. We carried out the data analyses using the MSD discovery workbench software.

### ELISA for detection of MMP-9

We obtained murine MMP-9 detection kits from R&D System and analyzed levels of total MMP-9 in plasma and brains followed the manufacturer’s instructions. We recorded the results by a Biotek Synergy H1 Hybrid plate reader (wavelength = 450 nm).

### Statistical analyses

We conducted statistical analyses with PRISM 7 software (GraphPad Software, La Jolla, CA). One-way analysis of variance followed by post hoc test was used for the analysis of data above two groups. The Brown–Forsythe test was performed for equal population variances among multiple comparisons. Student’s *t*-test was used for the analysis of data between two groups. G*Power v.3.1.9.2 was used to decide power for the animal study. Fisher’s exact test was used to determine the difference of mortality between groups. All measurements were taken from distinct samples. Statistical significance was set at *p* < 0.05 (two-sided).

### Reporting summary

Further information on research design is available in the Nature Research Reporting Summary linked to this article.

## Supplementary information

Supplementary Information

Reporting summary

## Data Availability

Source data are provided as a Source Data file with this paper. The data that support the findings of this study are available from the corresponding author (X.R.) upon reasonable request. Source data are provided with this paper.

## Source data

source data
