# Author Correction: Blood substitution therapy rescues the brain of mice from ischemic damage

**DOI:** 10.1038/s41467-021-22615-0

**Published:** 2021-05-13

**Authors:** Xuefang Ren, Heng Hu, Imran Farooqi, James W. Simpkins

**Affiliations:** 1 Department of Neuroscience, West Virginia University, Morgantown, WV USA; 2 Department of Microbiology, Immunology and Cell Biology, School of Medicine, West Virginia University, Morgantown, WV USA; 3 Experimental Stroke Core, Center for Basic and Translational Stroke Research, Rockefeller Neuroscience Institute, West Virginia University, Morgantown, WV USA; 4 Present Address: Erma Building Room 109, Morgantown, WV USA; 5 Present Address: 64 Medical Center Drive, Morgantown, WV USA; 6 Present Address: EWMS Physical Medicine and Rehabilitation, Norfolk, VA USA; 7 Present Address: Erma Building Room 105, Morgantown, WV USA

**Keywords:** Neuroimmunology, Stroke

Correction to: *Nature Communications* 10.1038/s41467-020-17930-x, published online 25 August 2020.

The original version of this Article included an incorrect Source Data file, in which raw data underlying Fig. 6c (ipsilateral Stroke + BR group), 6g (ipsilateral Stroke + BR group), 6i (ipsilateral Stroke + BR group and contralateral Stroke group), and 7d (contralateral MMP-9 levels) contained errors. These errors arose during transfer of data between file formats during the data analysis.

The HTML has been updated to include a corrected version of the Source Data file; the original incorrect version of the Source Data file can be found as Supplementary Information associated with this Correction.

The original version of this Article contained errors in Fig. 6c (ipsilateral Stroke + BR group), 6g (ipsilateral Stroke + BR group), 6i (ipsilateral Stroke + BR group and contralateral Stroke group). These errors arose during transfer of data between file formats during the data analysis.

The correct version of Fig. 6 is:

which replaces the previous incorrect version:

The original version of the Article contained errors in Fig. 7d (contralateral MMP-9 levels). These errors arose during transfer of data between file formats during the data analysis.

The correct version of Fig. 7 is:

which replaces the previous incorrect version:

This has been corrected in both the PDF and HTML versions of the Article.

## Supplementary information

Supplementary information

