# Supplementary material for: Blood substitution therapy rescues the brain of mice from ischemic damage
Source: Nat Commun. 2020 Aug 25;11:4078. doi: 10.1038/s41467-020-17930-x (PMC7447645; doi:10.1038/s41467-020-17930-x)
Supplement: Supplementary file 1 — Supplementary Information [file 41467_2020_17930_MOESM1_ESM.pdf]

Supplementary Information for

Blood Substitution Therapy Rescues the Brain of Mice from Ischemic Damage

Ren *et al*.

Supplementary Methods

Study Approval: The ARRIVE guidelines were followed and Institutional Animal Care and Use Committee at West Virginia University (WVU) approved criteria for procedures prior to experimentation.

Animals: We used male 8~12 months old C57/BL6J mice (above 30g; Jackson's Laboratory, ME) for recipients and male 3~6 months old C57/BL6J mice for blood donors. We numbered the animals and allocated them into groups using a simple randomization of excel-generated random numbers. To avoid biases, we also assured that different treatments were performed on the same day.

ELISA for Detection of MMP-2: We obtained murine MMP-2 detection kits from R&D System and analyzed levels of total MMP-2 in plasma followed the manufacturer's instructions. We recorded the results by a Biotek Synergy H1 Hybrid plate reader (wavelength = 450nm).

Physiological analysis: We anesthetized mice and cannulated femoral artery for measurements of arterial blood gases (blood analysis system, IRMA TRUPOINT, Vetlab Supply), blood glucose (Glucometer Microlet 2, Bayer), and blood pressure (pressure monitor BP-1, World Precision Instruments, FL). Repeated measurements were made throughout the blood replacement period.

Supplementary Figures

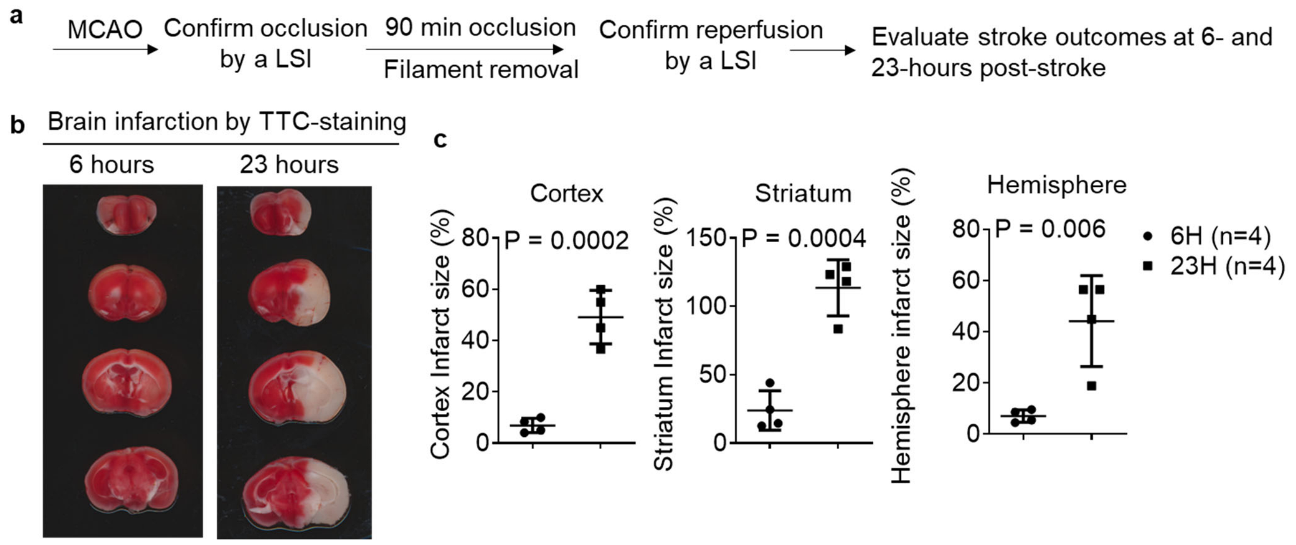

Supplementary Figure 1. Stroke infarction evolves over time following a transient middle cerebral artery occlusion (tMCAO).

(**a**) Experimental Design. (**b**) Representative TTC-stained coronal sections indicate brain infarction of mice. Viable brain tissue is stained red and infarct brain tissue remains white by TTC-staining. The filaments in MCAs were removed after 90 min occlusion. (**c**) Quantified infarct volumes revealed larger infarction from stroke mice at 23-hours (squares, n = 4) compared to 6-hours (circles, n = 4). Data were presented as means ± SD; P-values were calculated using two-tailed grouped analyses by Student's t test. Source data are provided as a Source Data file.

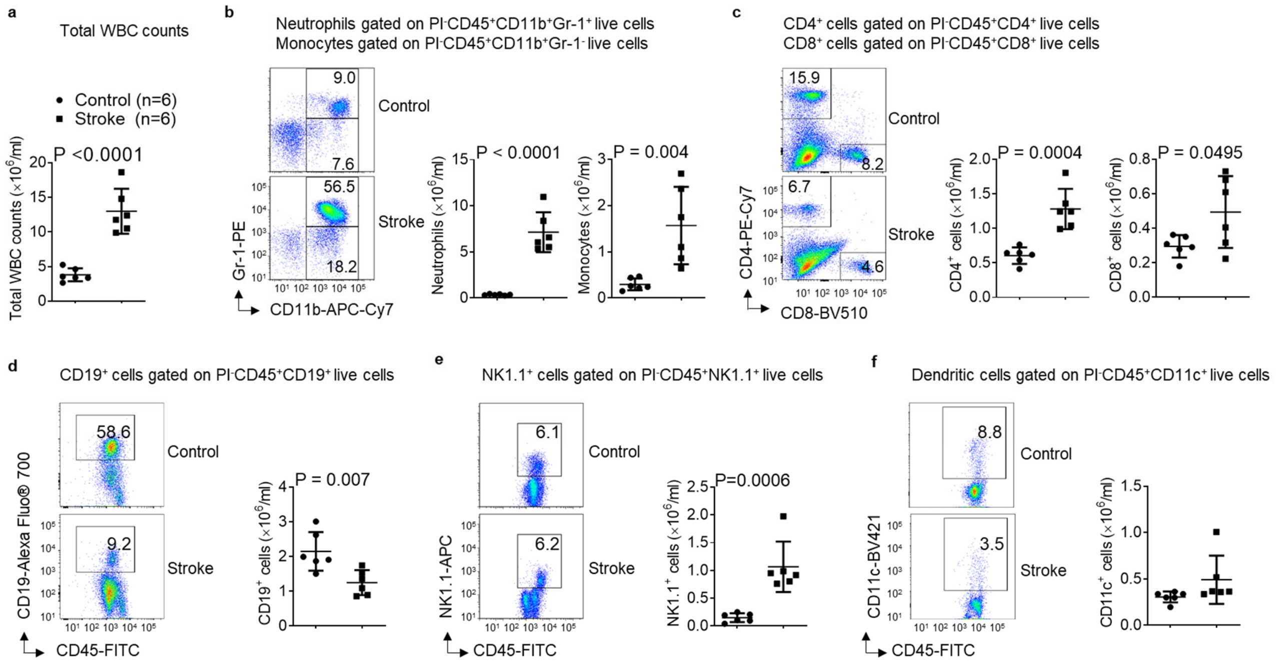

Supplementary Figure 2. A stroke alters cellular profiles in the blood at 6-hours stroke induction.

Blood obtained from control (circles, n = 6) and stroke mice (underwent tMCAO for 90 minutes) (squares, n = 6) were analyzed. (**a**) Stroke increased total white blood cell (WBC) counts in the blood. (**b**) Stroke increased total neutrophils and monocytes in the blood. Neutrophils were gated on PI$^-$CD45$^+$CD11b$^+$Gr1$^+$ live cells and monocytes were gated on PI$^-$CD45$^+$CD11b$^+$Gr1$^-$ live cells for the analyses detected by flow cytometry. (**c**) Stroke increased total CD4$^+$ and CD8$^+$ cells in the blood. CD4$^+$ cells were gated on PI$^-$CD45$^+$CD4$^+$ live cells and CD8$^+$ cells were gated on PI$^-$CD45$^+$CD8$^+$ live cells for the analyses. (**d**) Stroke reduced total CD19$^+$ B-cells in the blood. B cells were gated on PI$^-$CD45$^+$CD19$^+$ live cells for the analyses. (**e**) Stroke increased total NK1.1$^+$ cells in the blood. NK1.1$^+$ cells were gated on PI$^-$CD45$^+$NK1.1$^+$ live cells. (**f**) Stroke did not change total number of CD11c$^+$ dendritic cells in the blood. Data were presented as means ± SD; P-values were calculated using two-tailed grouped analyses by Student's t test. Source data are provided as a Source Data file.

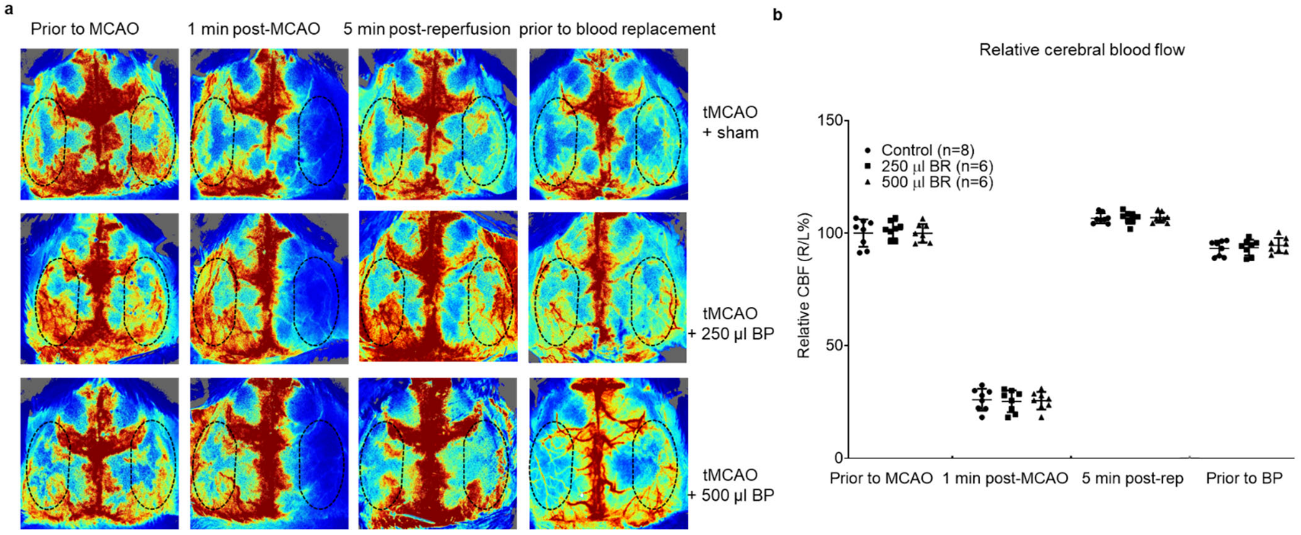

Supplementary Figure 3. Relative cerebral blood flow was not significantly different among randomized groups.

Mice underwent tMCAO for 90 minutes. The cerebral blood flow was recorded at four time points - prior to MCAO, 1 min post-MCAO, 5 min post-reperfusion, and prior to blood-replacement by Laser Speckle Imager. The occlusion and reperfusion were confirmed by Laser Speckle Imager then mice were randomized into three groups: sham control (circles, n = 8), 250μl (squares, n = 6), or 500μl (triangles, n = 6) of blood from naive healthy donor mice (3 months old). (**a**) Representative images of cerebral blood flow (CBF) from Laser Speckle Imager. MCA territories are outlined with dashed lines. (**b**) Quantified relative CBF (R/L) (ratio of CBF in right MCA territory: CBF in left MCA territory). The data confirmed that the MCAO reduced 70~80% relative CBF at the post-tMCAO time point. The reperfusion fully recovered the relative CBF at 5 min post-reperfusion and prior to blood-replacement in all stroke mice; however, no significant differences were detected among randomized groups of mice. Data were presented as means ± SD; One-way ANOVA followed by post-hoc Fisher's unprotected least significant difference multiple comparison tests. Source data are provided as a Source Data file.

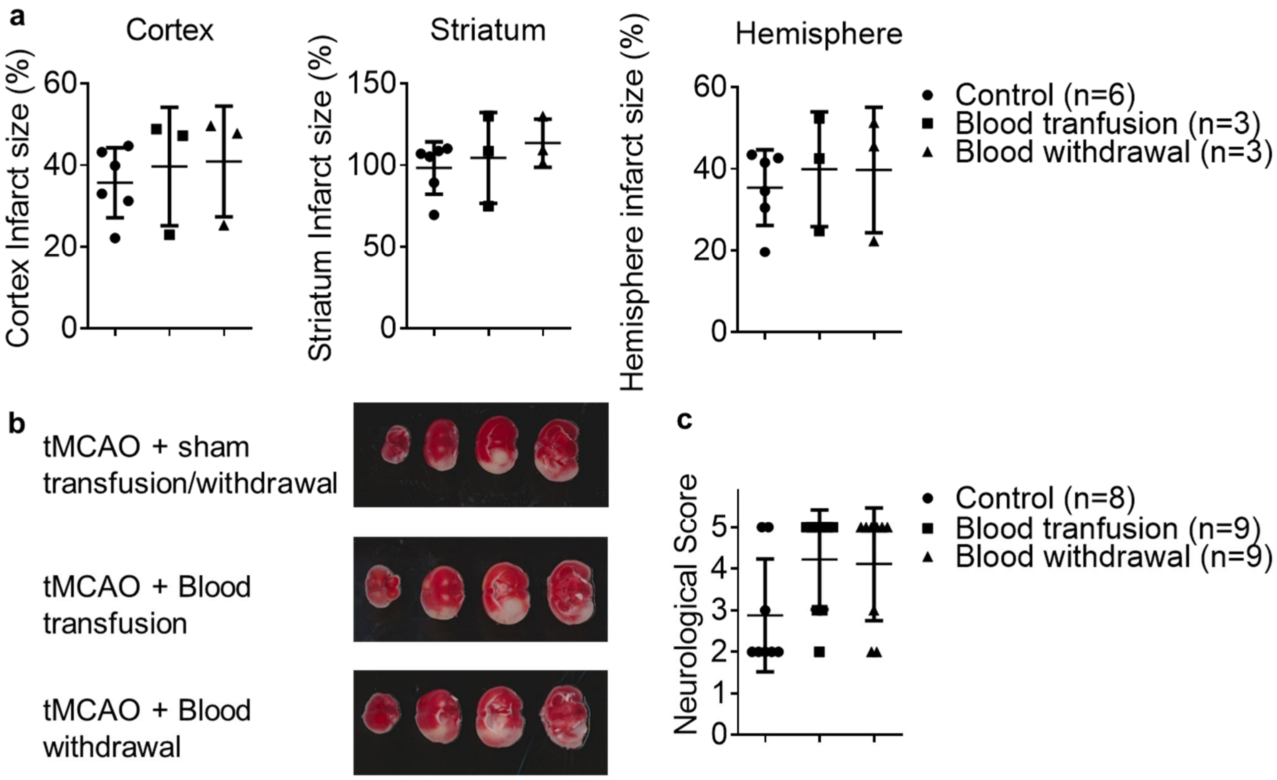

Supplementary Figure 4. Blood transfusion or blood withdrawal alone does not protect stroke outcomes.

Mice underwent tMCAO for 90 minutes were randomized to three groups: sham transfusion/withdrawal procedure group, transfusion of 500μl blood group, and withdrawal of 500μl blood group. Brain infarct volumes were measured at 23 hours after ischemia induction. (**a**) Quantified infarct volumes were not significantly changed by the blood transfusion or the blood withdrawal alone. Sham transfusion/withdrawal procedure group (circles, n=6), transfusion of 500μl blood group (squares, n=3), and withdrawal of 500μl blood group (triangles, n=3). (**b**) Representative TTC-stained coronal sections. (**c**) Neurological deficits were not significantly different in three groups. Sham transfusion/withdrawal procedure group (circles, n=8), transfusion of 500μl blood group (squares, n=9), and withdrawal of 500μl blood group (triangles, n=9). Data were presented as means ± SD; One-way ANOVA followed by post-hoc Fisher's unprotected least significant difference multiple comparison tests. 2 mice died in control group, 6 mice died in blood transfusion alone group, and 6 mice died in blood withdrawal alone group. Source data are provided as a Source Data file.

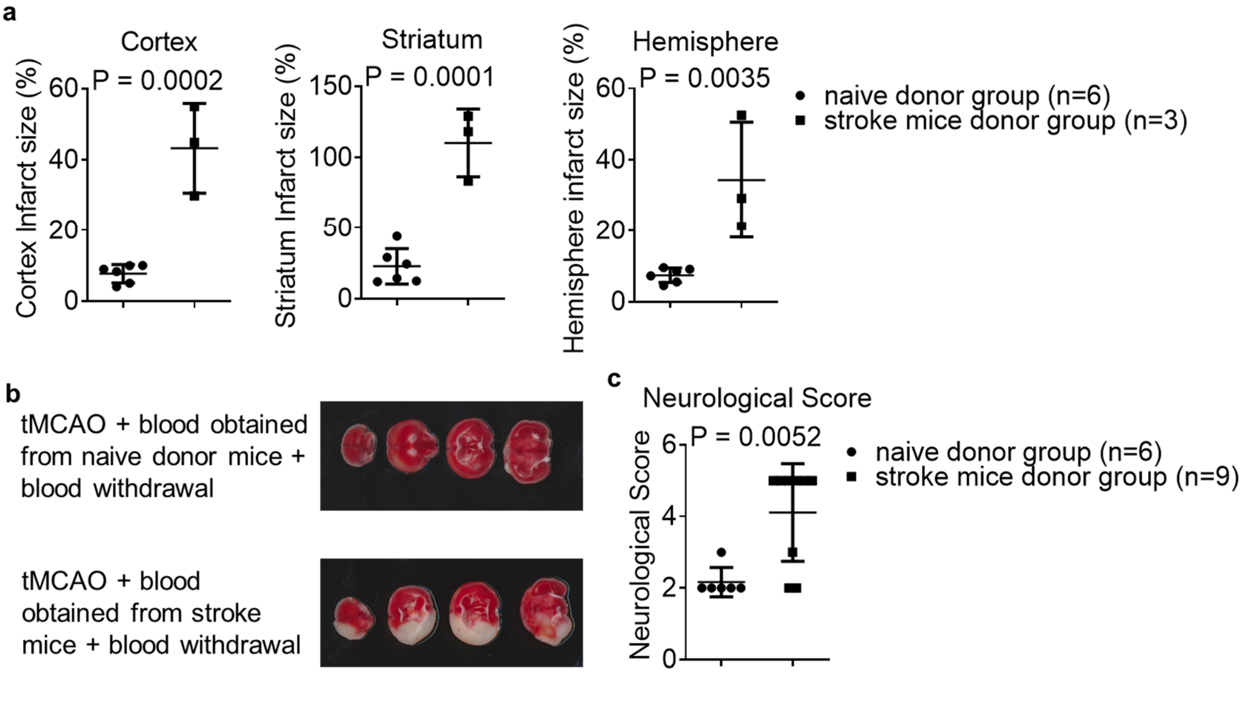

Supplementary Figure 5. Blood obtained from stroke mice does not protect stroke outcomes.

Mice underwent tMCAO for 90 minutes and randomized to two groups: transfusion of 500μl blood obtained from naive donor mice (circles) and transfusion of 500μl blood obtained from stroke mice (received permanent MCAO for 6 hours) (squares). The same volume of blood was withdrawn from the recipient stroke mice during blood transfusion. Brain infarct volumes were measured at 23 hours after ischemia induction. (**a**) Blood obtained from stroke mice had significantly larger infarct volume in cortex, striatum, and total hemisphere than blood obtained from naive donors in stroke mice received blood replacement. (**b**) Representative TTC–stained coronal sections. (**c**) Blood obtained from stroke mice had significantly worse neurological deficits than blood obtained from naive donor mice in stroke mice received blood replacement. Data were presented as means ± SD; P-values were calculated using two-tailed grouped analyses by Student's t test. N=3 or 6 per group for stroke infarction, n=6 or 9 per group for neurological deficits; No mice died in blood obtained from naïve donor group but 6 mice died in blood obtained from stroke mice group. Source data are provided as a Source Data file.

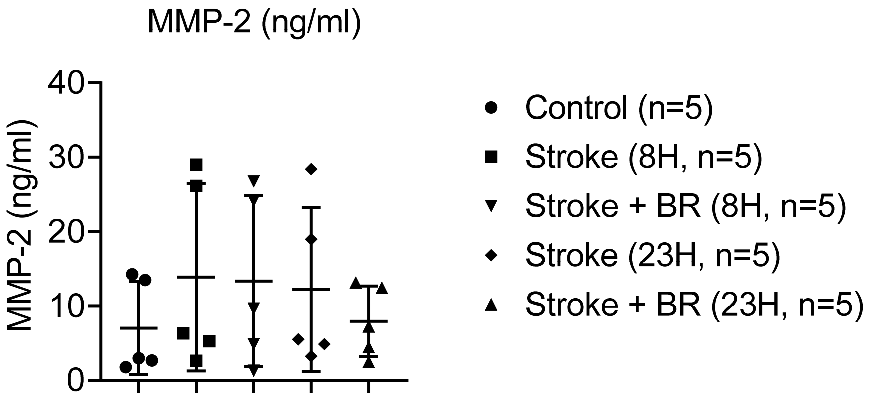

Supplementary Figure 6. Blood replacement therapy does not significantly change levels of MMP-2 in the plasma of stroke mice.

Mice (8~12 months old males) underwent tMCAO for 90 minutes. The occlusion and reperfusion were confirmed by a laser speckle imager (LSI). Stroke mice were then randomized into two groups: a stroke only group and the blood replacement (BR) group receiving 500μl of blood from healthy donors. Blood was replaced into stroke mice at 6.5~7 hours post-stroke. Brains were perfused and analyzed at 8- and 23-hours after ischemia induction. Control (circles, n=5), stroke mice analyzed at 8 hours post-stroke (squares, n=5), stroke mice with BR therapy analyzed at 8 hours post-stroke (down-pointing triangles, n=5), stroke mice analyzed at 23 hours post-stroke (diamonds, n=5), stroke mice with BR therapy analyzed at 23 hours post-stroke (up-pointing triangles, n=5). The levels of MMP-2 in plasma were not significantly changed by BR therapy at 8- and 23-hours post-stroke. Data were presented as means ± SD; One-way ANOVA followed by post-hoc Fisher's unprotected least significant difference multiple comparison tests. N=5 per group. Source data are provided as a Source Data file.

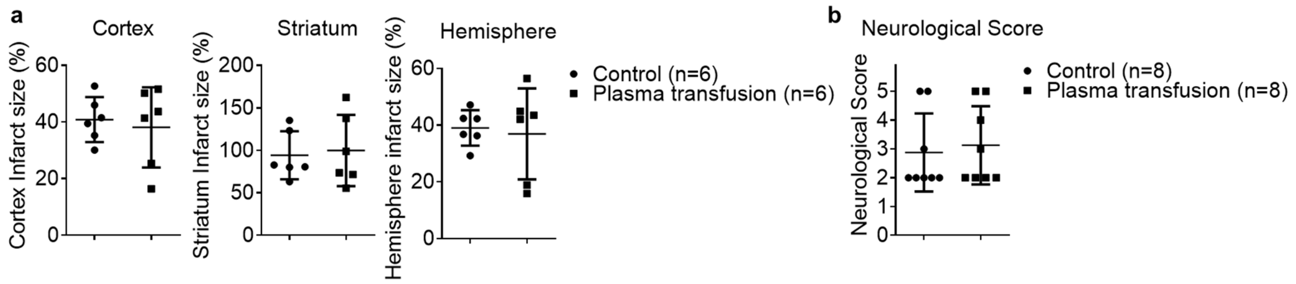

Supplementary Figure 7. Plasma transfusion does not protect stroke outcomes.

Mice underwent tMCAO for 90 minutes and randomized to two groups: transfusion of 500µl plasma obtained from naive donor mice and a plain stroke group. The same volume of blood was withdrawn from the recipient stroke mice during plasma transfusion. (**a**) Plasma transfusion did not significantly change infarct volume in cortex, striatum, and total hemisphere compared to control mice. Control group (circles, n=6), plasma group (squares, n=6). (**b**) Plasma transfusion did not significantly change neurological deficits compared to control mice Control group (circles, n=8), plasma group (squares, n=8). Data were presented as means ± SD; Two-tailed grouped analyses by Student’s t test. Source data are provided as a Source Data file.

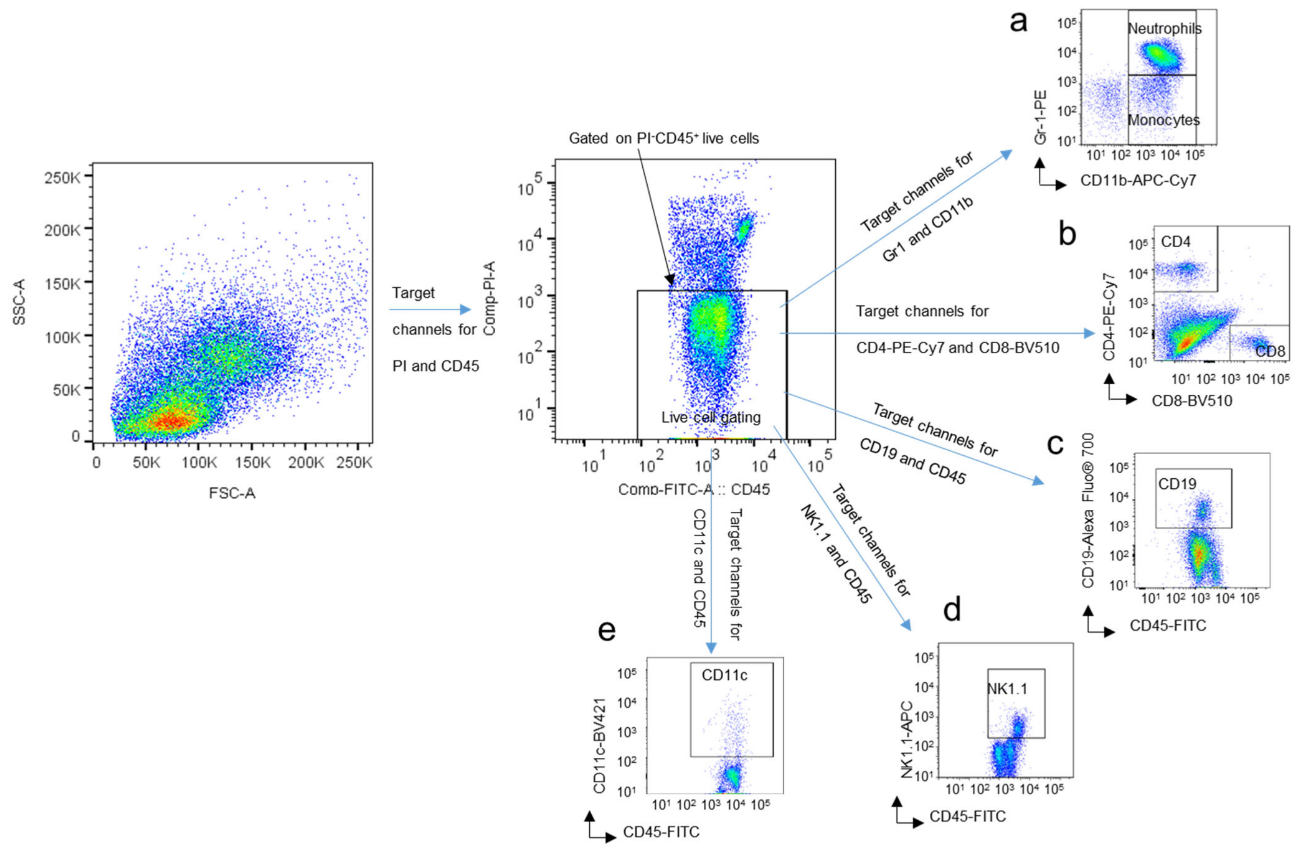

Supplementary Figure 8. Flow cytometry gating strategies for white blood cells. Live cells were gated on propidium Iodide (PI) negative $^{(-)}$ CD45 positive $^{(+)}$ populations. (**a**) Gating strategy to determine the percentages of neutrophils and monocytes in Fig. 4c and supplementary Fig. 2b. (**b**) Gating strategy to determine the percentages of CD4 cells and CD8 cells in Fig. 4f and supplementary Fig. 2c. (**c**) Gating strategy to determine the percentages of CD19 B-cells in Fig. 4i and supplementary Fig. 2d. (**d**) Gating strategy to determine the percentages of NK1.1 cells in Fig. 4k and supplementary Fig. 2e. (**e**) Gating strategy to determine the percentages of Dendritic cells in supplementary Fig. 2f.

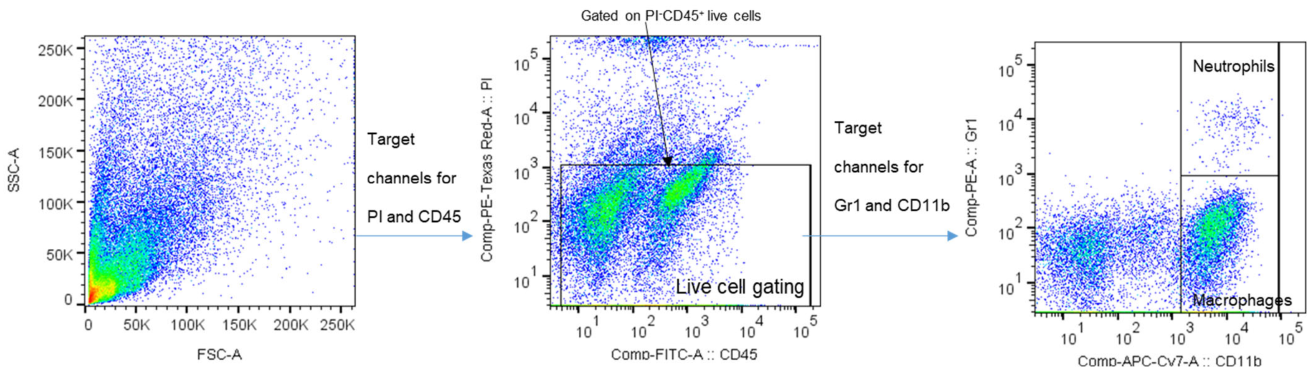

Supplementary Figure 9. Flow cytometry gating strategies for brain cells. Live cells were gated on propidium Iodide (PI) negative $^{(-)}$ CD45 positive $^{(+)}$ populations. Gating strategy to determine the percentages of neutrophils and macrophages in Fig. 6d & e.

Supplementary Table 1. Physiological parameters at pre-, mid- and post-blood replacement in stroke mice.

Variables	Control (n=4)			Blood replacement (BR) (n=4)		
	Pre-BR	Mid-BR	Post-BR	Pre-BR	Mid-BR	Post-BR
PaO2 (mm Hg)	165 ± 15	154 ± 14	171 ± 20	167 ± 11	168 ± 15	175 ± 23
Blood glucose (mg/dL)	136 ± 17	128 ± 12	130 ± 18	138 ± 15	131 ± 17	135 ± 20
pH value	7.40 ± 0.07	7.41 ± 0.05	7.37 ± 0.06	7.40 ± 0.04	7.42 ± 0.07	7.38 ± 0.09
PaCO2 (mm Hg)	16 ± 3	21 ± 4	21 ± 9	17 ± 6	21 ± 8	21 ± 5
Temperature (°C)	36.8 ± 0.7	36.9 ± 0.5	37.0 ± 0.4	36.6 ± 0.4	37.1 ± 0.5	37.1 ± 0.3
Mean blood pressure (mm Hg)	67 ± 5	79 ± 7	81 ± 9	68± 7	80 ± 4	83 ± 7

Source data are provided as a Source Data file.

Supplementary Table 2. Antibodies used in the study.

Antibodies	Clone	Catalog number	Lot number	Vendors
CD45-FITC	30-F11	11-0451-85	4277451	eBioscience
Gr-1-PE	1A8	551461	3337642	BD Pharmingen™
CD11b-APC-Cy7	M1/70	557657	5351540	BD Pharmingen™
CD4-PE-Cy7	GK1.5	563933	5198694	BD Pharmingen™
CD8-BV510	53-6.7	563068	6028778	BD Horizon™
CD19-Alexa Fluor®700	1D3	557958	6078746	BD Pharmingen™
NK1.1-APC	PK136	17-5941-81	E07336-1631	eBioscience
