# Supplementary material for: Blood substitution therapy rescues the brain of mice from ischemic damage
Source: Nat Commun. 2020 Aug 25;11:4078. doi: 10.1038/s41467-020-17930-x (PMC7447645; doi:10.1038/s41467-020-17930-x)
Supplement: Supplementary file 2 — Reporting summary [file 41467_2020_17930_MOESM2_ESM.pdf]

nature research

Corresponding author(s): Xuefang Ren & James W. Simpkins

Last updated by author(s): Jul 14, 2020

Reporting Summary

Nature Research wishes to improve the reproducibility of the work that we publish. This form provides structure for consistency and transparency in reporting. For further information on Nature Research policies, see Authors & Referees and the Editorial Policy Checklist.

Statistics

For all statistical analyses, confirm that the following items are present in the figure legend, table legend, main text, or Methods section.

n/a	Confirmed	
☐	☒	The exact sample size (*n*) for each experimental group/condition, given as a discrete number and unit of measurement
☐	☒	A statement on whether measurements were taken from distinct samples or whether the same sample was measured repeatedly
☐	☒	The statistical test(s) used AND whether they are one- or two-sided<br>*Only common tests should be described solely by name; describe more complex techniques in the Methods section.*
☒	☐	A description of all covariates tested
☐	☒	A description of any assumptions or corrections, such as tests of normality and adjustment for multiple comparisons
☐	☒	A full description of the statistical parameters including central tendency (e.g. means) or other basic estimates (e.g. regression coefficient) AND variation (e.g. standard deviation) or associated estimates of uncertainty (e.g. confidence intervals)
☐	☒	For null hypothesis testing, the test statistic (e.g. *F*, *t*, *r*) with confidence intervals, effect sizes, degrees of freedom and *P* value noted<br>*Give P values as exact values whenever suitable.*
☒	☐	For Bayesian analysis, information on the choice of priors and Markov chain Monte Carlo settings
☒	☐	For hierarchical and complex designs, identification of the appropriate level for tests and full reporting of outcomes
☒	☐	Estimates of effect sizes (e.g. Cohen's *d*, Pearson's *r*), indicating how they were calculated

Our web collection on statistics for biologists contains articles on many of the points above.

Software and code

Policy information about availability of computer code

Data collection	BD FACS Diva version 8.0 (build 2013 07 02 02 11) Firmware 1.4
Data analysis	PRISM 7 software (code: GPS-1283310-THPP-08A40), G*Power v.3.1.9.2 (free online version), Flowjo version 10 software (code: S1GNcGa1Tg1Z54Ux); Image J (NIH online free version)

For manuscripts utilizing custom algorithms or software that are central to the research but not yet described in published literature, software must be made available to editors/reviewers. We strongly encourage code deposition in a community repository (e.g. GitHub). See the Nature Research guidelines for submitting code & software for further information.

Data

Policy information about availability of data

All manuscripts must include a data availability statement. This statement should provide the following information, where applicable:

- Accession codes, unique identifiers, or web links for publicly available datasets
- A list of figures that have associated raw data
- A description of any restrictions on data availability

Figures 1, 3-8, Supplementary figures 1-7, Supplementary Table 1 are associated with the Source Data file. The raw data that support the findings of this study are available from the corresponding authors upon reasonable request. No restrictions for data availability.

Field-specific reporting

Please select the one below that is the best fit for your research. If you are not sure, read the appropriate sections before making your selection.

☒ Life sciences ☐ Behavioural & social sciences ☐ Ecological, evolutionary & environmental sciences

For a reference copy of the document with all sections, see nature.com/documents/nr-reporting-summary-flat.pdf

Life sciences study design

All studies must disclose on these points even when the disclosure is negative.

Sample size	G*Power v.3.1.9.2 was used to decide power based upon the pilot animal study.
Data exclusions	Five mice were excluded in the study following our previous exclusion criteria: 3 mice (1 mouse in sham blood-replacement control group and 2 mice in blood-replacement group of blood obtained from stroke mice) because of subarachnoid hemorrhage, 2 mice (prior to group randomization) because the Laser Speckle Imager did not detect 70% reduction of CBF after MCAO.
Replication	The data are very well repeatable. We have done several studies related to the project and the results repeated showing the same trend. In this paper, the results in Fig. 1 , Fig. 3, Fig. 8, and supplemental Fig. 5 obtained from individual experiment, all demonstrated that blood replacement protected brains from stroke damage. In addition, we haven't reported the data from recipient stroke mice with a wide range of ages, which have demonstrated all stroke mice are protected from stroke damage by blood replacement therapy. We will report the data in the future once more studies are performed.
Randomization	We numbered the animals and allocated them into groups using a simple randomization of excel-generated random numbers. To avoid biases, we also assured that different treatments were performed on the same day.
Blinding	The experimenters were blinded to the treatments for data collection and data analysis. The surgeon who knew the treatment groups was not assigned to data collection or analyses.

Reporting for specific materials, systems and methods

We require information from authors about some types of materials, experimental systems and methods used in many studies. Here, indicate whether each material, system or method listed is relevant to your study. If you are not sure if a list item applies to your research, read the appropriate section before selecting a response.

Materials & experimental systems

n/a	Involved in the study	
☐	☒	Antibodies
☒	☐	Eukaryotic cell lines
☒	☐	Palaeontology
☐	☒	Animals and other organisms
☒	☐	Human research participants
☒	☐	Clinical data

Methods

n/a	Involved in the study	
☒	☐	ChIP-seq
☐	☒	Flow cytometry
☒	☐	MRI-based neuroimaging

Antibodies

Antibodies used	The information is provided in supplementary Table 2.
Validation	Validation statements are provided on the manufacturers' website, relevant citations, and antibody profiles in online databases.

Animals and other organisms

Policy information about studies involving animals; ARRIVE guidelines recommended for reporting animal research

Laboratory animals	Male 8~12 months old C57/BL6J mice (above 30g; Jackson's Laboratory, ME) for recipients and male 3~6 months old C57/BL6J mice for blood donors.
Wild animals	The study did not involve wild animals.
Field-collected samples	The study did not involve samples collected from the field.
Ethics oversight	Institutional Animal Care and Use Committees at West Virginia University approved criteria for procedures prior to experimentation.

Note that full information on the approval of the study protocol must also be provided in the manuscript.

Flow Cytometry

Plots

Confirm that:

☒ The axis labels state the marker and fluorochrome used (e.g. CD4-FITC).

☒ The axis scales are clearly visible. Include numbers along axes only for bottom left plot of group (a 'group' is an analysis of identical markers).

☒ All plots are contour plots with outliers or pseudocolor plots.

☒ A numerical value for number of cells or percentage (with statistics) is provided.

Methodology

Sample preparation	The procedures are detailed in the method section. Red blood cells were lysed with lysis buffer then cells were washed with buffer (PBS with 0.5%BSA and 0.02% sodium azide) three times, and stained with antibodies.
Instrument	BD FACS LSRFortesa (twelve fluorochromes, three-laser system, BD Biosciences) with FACS Diva version 8.0 software (BD Biosciences)
Software	BD FACS Diva version 8.0 ((build 2013 07 02 02 11) was used to collect the flow cytometry data. Flowjo version 10 software was used to analyze the data.
Cell population abundance	Cell population abundances varies on FSC/SSC plot per treatment. Therefore, CD45+PI- gating strategy was further applied. All white blood cell populations were 100% CD45+PI- population.
Gating strategy	Preliminary FSC/SSC plots were gated on lymphocyte, granulocyte, and monocyte populations. CD45+PI- gating strategy was further applied to include white blood cell population and exclude dead cells. Isotype IgG controls were used to define positive and negative staining populations.

☒ Tick this box to confirm that a figure exemplifying the gating strategy is provided in the Supplementary Information.
